# Fracture of a Metal-Backed Ceramic Liner After Total Hip Arthroplasty: A Case Report

**DOI:** 10.7759/cureus.41824

**Published:** 2023-07-13

**Authors:** William Diciurcio, Rex W Lutz, Eric B Smith, Gregory K Deirmengian

**Affiliations:** 1 Orthopaedic Surgery, Jefferson Health New Jersey, Stratford, USA; 2 Orthopaedic Surgery, Rothman Orthopaedic Institute, Philadelphia, USA

**Keywords:** total hip arthroplasty, ceramic fracture, ceramic bearings, failed total hip, ceramic-on-ceramic

## Abstract

Total hip arthroplasty (THA) is a common procedure that has become increasingly prevalent in a younger patient population. With improvements in prostheses and materials, the survivorship of implants has increased. Historically, the excellent wear characteristics of ceramic-on-ceramic (CoC) implants made them an appealing choice compared to other bearing options. Yet, the potential benefits of the bearing longevity related to the wear characteristics have been combated by their unique causes of failure such as implant fracture and squeaking. Metal-backed ceramic liners were developed to minimize impingement-related chipping at the periphery of the implant that may propagate to catastrophic implant fracture. We report a case involving a fracture of a metal-backed ceramic liner that presented with months of pain and crepitus with no overt signs of fracture on imaging.

## Introduction

Total hip arthroplasty (THA) is one of the most common orthopedic surgeries that was historically reserved for elderly patients with end-stage osteoarthritis. Improvements in the material properties of arthroplasty components have led to their more widespread use in a younger, more active patient population [1]. The survivorship of THAs is excellent, with quite a high survivorship at 20 years [2-3]. Traditionally, the most common THA bearing was a cobalt-chromium femoral head articulating with a polyethylene liner; however, wear debris generated from conventional polyethylene led to osteolysis and implant failure [3]. In an effort to improve the lifespan of the implant, other bearing combinations were developed.

In the 1970s, ceramic-on-ceramic (CoC) bearings were first introduced. Alumina ceramics are extremely hard, scratch-resistant, and have a low coefficient of friction, leading to improved wear rates compared to other available bearing materials [4]. The advantages of alumina CoC bearings are apparent; however, there are a few disadvantages. Implant fracture and chipping are the specific complications resulting from the high Young’s modulus of ceramic [4]. These implants’ lifetime fracture risk is estimated at 0.03-0.05% for the femoral head and 0.013-0.017% for the acetabular liner. To decrease this risk, ceramic liners were designed with a partial or complete metal backing, decreasing the risk of chipping at the periphery due to impingement, which may lead to subsequent catastrophic fractures [4]. We report the case of a fracture of a partially metal-backed ceramic acetabular liner that presented with no radiographic evidence of fracture.

## Case presentation

A 66-year-old female with a past medical history only of hypertension presented 12 years after an uncomplicated right THA for primary osteoarthritis with a CoC implant (Smith and Nephew, Memphis, TN). She reported several months of right posterolateral hip pain and prosthetic noise. The patient denied any trauma, falls, or inciting events. She described her pain as a posterolateral grinding/popping sensation, which was exacerbated with walking and activities and rated 6/10 on a Likert scale. On initial workup, radiographs showed a stable THA with no overt signs of failure. A CT scan was ordered for further evaluation, demonstrating no signs of prosthetic failure. An infectious workup was conducted when she first presented, including C-reactive protein (CRP) (0.10 mg/dl) and erythrocyte sedimentation rate (ESR) (23). Because an indication for revision was not evident, revision surgery was not recommended.

**Figure 1:**
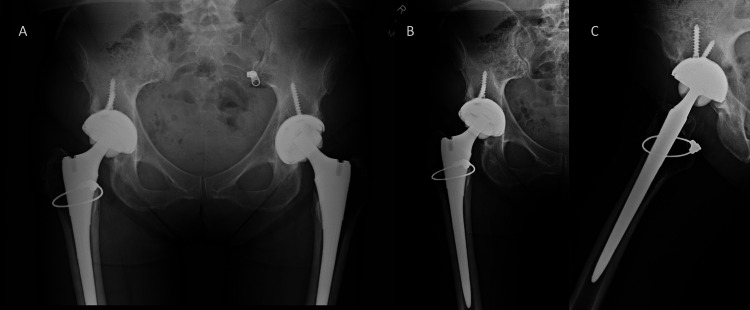
Preoperative anteroposterior (A) radiograph of the pelvis and anteroposterior (B) and lateral (C) radiograph of the hip showing a well-fixed and well-aligned right total hip arthroplasty with no signs of failure

The patient ultimately sought a second opinion months later after continuing to have pain. On examination, the patient was 5 feet 5 inches tall and weighed 140 pounds. She had a positive Stinchfield test [5] and pain with the range of motion of the right hip. In addition, crepitus was palpated about the trochanteric area with ambulation. Radiographs at that time showed a well-aligned, stable right CoC THA (Figure 1).

Since she failed extensive conservative treatment, she was offered a revision to explore potential sources of her mechanical crepitus and pain.

**Figure 2:**
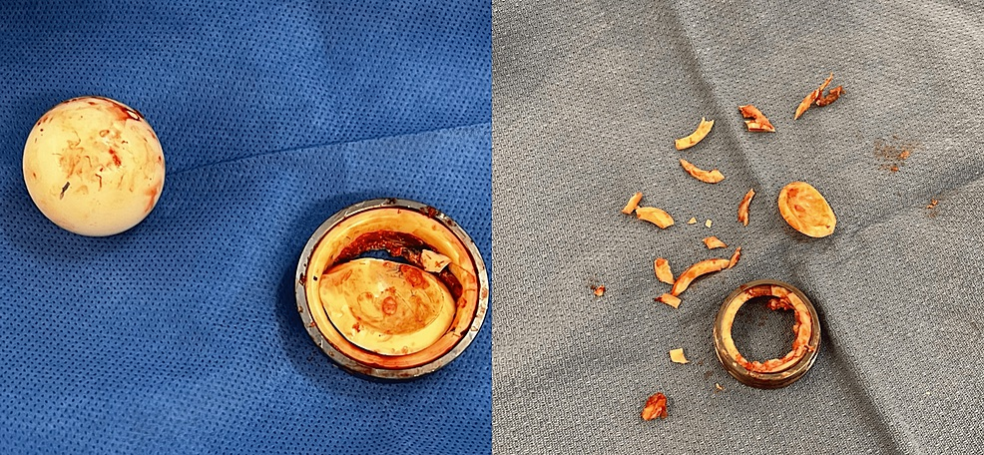
Clinical photographs demonstrating the fractured ceramic liner

At the time of surgery, a modified Hardinge approach was used using the existing lateral scar. The patient had the primary hip replacement through a modified Hardinge approach, which was also the approach the revision surgeon typically used. The cerclage wire was removed, the hip was dislocated, and the femoral head was removed and found to be undamaged. On intraoperative inspection, the femoral stem was found to be well-fixed. The acetabular component was exposed and the ceramic liner was noted to be fractured in a nondisplaced manner, explaining the lack of imaging findings preoperatively. Upon dislodging the metal-backed liner from the acetabular shell, the ceramic fracture displaced into multiple fragments that remained contained within the shell (Figure 2).

**Figure 3:**
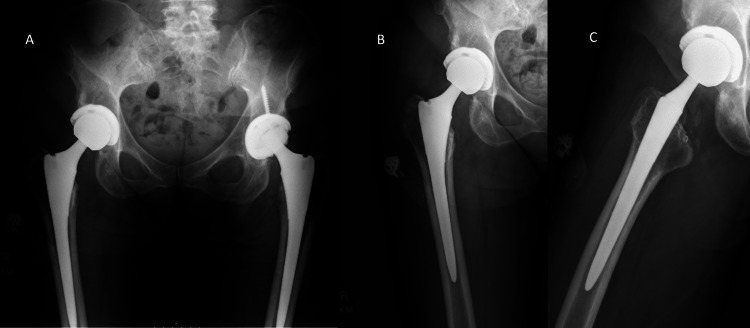
Postoperative anteroposterior (A) radiograph of the pelvis and anteroposterior (B) and lateral (C) radiograph of the hip showing a right total hip revision after bearing exchange

The liner and all visible fragments were removed. After screw removal, the acetabular shell was found to be well-fixed and well-positioned and so it was retained. A polyethylene liner was impacted, and a 36 mm Oxinium femoral head was impacted onto the femoral trunion. Reduction of the hip and trialing showed excellent stability. Postoperatively, the patient was weight-bearing as tolerated with hip precautions and instructed to continue her at-home exercises and therapy. At six months postoperatively, the patient reported no pain, an excellent overall functional outcome, and no more crepitus. Radiographs showed a well-aligned and well-fixed right THA (Figure 3).

## Discussion

The ceramic-on-ceramic bearing was developed to improve the longevity of hip replacements [6]. The main advantage of the hard-bearing construct is the lower rates of implant wear, theoretically allowing longer survivorship of the total hip components, especially in the younger population [6]. While there are known advantages of these hard-bearing designs, there are disadvantages that are unique to these implant designs. In CoC THA, the primary concern is the risk of implant fracture. According to various authors, the rate of fracture of CoC total hips is approximately 0.004%-0.19% [6,7]. Based on recent reviews, the survivorship of CoC THA is 93.8% to 97.0% at 10 years [6,7].

The factors that lead to ceramic liner fracture have been well-described. First, ceramic material properties can contribute to fracture, related to the inherent brittle nature of ceramic and manufacturing/design flaws [8]. Improvements in the manufacturing process, such as reducing the ceramic grain size (increasing its density), hot isostatic pressing, and laser marking, have all improved the durability of the ceramic liners [8,9]. Second, it has been thought that ceramic acetabular liner fractures initiate with chips and cracks at the periphery of the liner, caused by impingement. Technical errors, such as component retroversion, can lead to the acetabular liner impingement with the neck of the femoral stem. The impingement can cause a focal area of increased stress and lead to the liner fracturing [8]. To eliminate the impact of metal on ceramic impingement that may lead to chips that then propagate into catastrophic fractures, some ceramic liners were redesigned with a metal backing. Sleeved liners have increased burst strength by 50% compared to traditional liners [8]. Third, patient-related factors, such as anatomical variations, body habitus, and trauma, can contribute to ceramic THA components’ fractures. Finally, surgical techniques and intraoperative factors could lead to fractures. One study found that ceramic acetabular liners were not seated correctly 22.9% of the time and this may be underestimated [10]. 

There is no consensus regarding the management of ceramic liner fractures, and it is often a subject of debate. During revision THA, a total synovectomy and thorough irrigation are performed to remove all ceramic debris [11]. If ceramic debris is incompletely removed, third-body wear can ensue, leading to rapid early wear and subsequent catastrophic failure. Revision to a CoC bearing may be favored in revision surgery, as ceramic debris can interpose in the joint and cause accelerated wear and osteolysis if a polyethylene liner is used [8,11]. In our case, a zirconium oxide-bearing surface, Oxinium (Smith & Nephew, Memphis, TN), was used during the revision. The oxide layer produced through a thermally driven oxygen diffusion process is much harder and more scratch-resistant than the untreated alloy; however, alumina ceramic is harder and more scratch-resistant. Oxinium’s major potential advantage is the lower wear rates against traditional and cross-linked polyethylenes [4].

Two prior reported cases of fractures in metal-backed ceramic liners have been reported. Copp et al. reported a 63-year-old female with a history of lumbar lordosis with compensatory forward pelvis flexion; who underwent THA with a metal-backed ceramic liner. Five years postoperatively, the patient started to experience symptoms of clicking, grinding, and moderate pain in her hip with ambulation. Radiographs revealed a comminuted ceramic liner fracture [5]. The revision procedure involved removing all fragments, synovectomy, and inserting a 36mm ceramic Biolox femoral head and a polyethylene liner with a good outcome [5].

Su and Chotai reported a 60-year female who presented 27 months post-bilateral CoC THA with new-onset, atraumatic left hip clicking and grinding with hip extension. On exam, there was audible-palpable crepitus appreciated with the range of motion of the left hip. Radiographs demonstrated a fractured ceramic insert and an excessively anteverted socket. All fragments were removed, and a synovectomy was performed. The THA was revised to a metal head with a polyethylene liner. At the 4.5-year follow-up, the Harris Hip Score was 100, and there were no signs of osteolysis or polyethylene wear. In addition, to the intraoperative impingement noted, the patient’s acetabular cup was placed into excessive anteversion secondary to underlying acetabular dysplasia.​ The elevated metal rim decreased the arc of motion available before impingement occurred [8]. The common theme of these cases was impingement, which led to a liner fracture that was evident on preoperative radiographs.

The unique aspect of the case presented in this article was the lack of fracture identified on radiographs or advanced imaging. Furthermore, there was a lack of discrete intraoperative findings to explain the fracture. The patient denied any trauma or inciting events, and intraoperative findings during the revision revealed appropriately aligned implants. The ceramic head was not damaged, which points away from an axial load causing the fracture. The ceramic liner fractured despite the metal rim protecting the edge of the ceramic component from chipping. Also, the metal rim seemed to contain the ceramic fracture, prevent it from shattering, and contained the fragments within the shell once the liner was disengaged from the shell. This improved the ability to completely remove all fragments, minimizing the risk of catastrophic failure resulting from third-body wear.

We present a case of a fracture through a metal-backed ceramic acetabular liner that was not apparent on advanced imaging. The patient had a successful outcome following the revision THA. Physicians should be aware of the potential complications associated with metal-backed ceramic lines and be highly suspicious of implant fractures in patients with an otherwise negative workup.

## Conclusions

A ceramic-on-ceramic bearing carries a risk of fracture that can lead to catastrophic failure after hip replacement. A potential mechanism of this mode of failure is the chipping of the ceramic at the periphery due to component impingement, which can propagate into a fracture. Metal-backed liners have been developed to combat this mechanism. We report a case of a fracture of a partially metal-backed ceramic liner. The metal backing prevented displacement of the fracture and, as such, the fracture was not noted on preoperative imaging. In addition, the metal backing helped contain the broken fragment within the shell, allowing for easy removal of all ceramic fragments.
